# EEG Spectral Power Changes in Patients With Dysexecutive Syndrome Following Cognitive Intervention

**DOI:** 10.1002/brb3.70148

**Published:** 2024-11-22

**Authors:** Claire Lebely, Evelyne Lepron, Ines Bigarre, Caroline Hamery, Xavier De Boissezon, Sebastien Scannella

**Affiliations:** ^1^ Department of Physical Medicine and Rehabilitation University Hospital of Toulouse Toulouse France; ^2^ ToNIC, NeuroImaging Center University of Toulouse, Inserm, UPS Toulouse France; ^3^ Fédération ENAC ISAE‐SUPAERO ONERA Université de Toulouse Toulouse France

**Keywords:** acquired brain injury, cognitive training, EEG, power spectral analysis

## Abstract

**Background:**

Acquired brain injury (ABI) leads to cognitive deficiencies, alteration of brain activity associated with an increase in slow‐wave (delta and theta bands) power, and reduced fast‐wave (alpha, beta, and gamma bands) power. To compensate for the cognitive deficits that impact autonomy and quality of life, patients in a chronic phase can benefit from cognitive intervention.

**Objective:**

This study explores the effects of cognitive intervention on brain activity, measured by electroencephalography (EEG), and on executive functioning, assessed by the Test of Attentional Performance (TAP) battery.

**Method:**

We provided an ecological rehabilitation intervention, simulating real‐life tasks adapted for patients with chronic cognitive disorders. A single‐case experimental design (SCED) assessed patients' performance in terms of correct responses percentage (CRs) and reaction times (RTs), and EEG spectral powers before and 1 month after the intervention. The TAP tasks included working memory (WM), divided attention (DA), inhibition (GO), and flexibility (FL). EEG frequency powers were also measured during resting states.

**Results:**

One month after the intervention, significant improvements were observed in CRs and RTs for the FL task. Increases in all frequency band powers occurred during FL, WM, and DA tasks, except for alpha bands in DA. In the GO task, delta and gamma power also increased after the intervention. No significant changes were found during resting‐state EEG. The results of this open study, without a control group, are preliminary.

**Conclusion:**

The effects of the therapy are mostly reflected by changes in mental FL performance and altered EEG patterns during cognitive tasks, particularly in slow and fast‐frequency bands. We argue that cognitive intervention could amplify the compensatory mechanisms following brain damage and/or ease restoration mechanisms in the fast‐frequency activity bands. Further SCEDs or studies with control groups are needed to confirm these findings and the role of EEG biomarkers in rehabilitation.

## Graphical abstract

This study investigates the effects of cognitive rehabilitation on brain activity and executive functioning in patients with acquired brain injury (ABI). EEG recordings and performance on the Test of Attentional Performance (TAP) were analyzed before and one month after an ecological rehabilitation intervention. Results show significant improvements in executive functioning, particularly in mental flexibility (FL), as evidenced by faster reaction times and higher correct response rates. Additionally, EEG analysis revealed increased power in slow and fast frequency bands (delta, theta, gamma) during cognitive tasks, reflecting changes in brain activity associated with the intervention. These findings suggest cognitive rehabilitation may enhance compensatory mechanisms in brain function post‐injury.

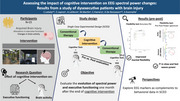

**Abbreviations**
ABI: acquired brain injury
ANR: French National Research Agency
CR: correct responses
DA: divided attention
DGA: Defence Procurement Agency
EEG: electroencephalography
FL: flexibility
GAS: Goal Attainment Scaling
GO: GoNoGo (inhibition task)
HD: high‐definition
mRS: modified Rankin score
NIHSS: National Institutes of Health Stroke Scale
rDLPFC: right dorsolateral prefrontal cortex
RT: reaction time
SCED: single‐case experimental design
T0: evaluation time before the start of the intervention
TAP: Test of Attentional Performance
TBI: traumatic brain injury
TF + 1: evaluation time 1 month after the end of the intervention
tRNS: transcranial random noise stimulation
WM: working memory

## 1 Introduction

Cognitive impairments are common sequelae following acquired brain injury (ABI) (van der Flier et al. 2018), whether from traumatic or vascular origin. They can lead to alterations in executive functions (EFs) (Kennedy et al. 2008) such as flexibility (FL), attention, or working memory (WM) (Clark and Manes 2004; Godefroy et al. 2010; Rabinowitz and Levin 2014). At the same time, understanding the neural mechanisms of EFs is also important to improve recovery after suffering from ABI. Over and above the observable behavioral changes, it has been shown that brain lesions lead to alterations in brain activity as measured by electroencephalography (EEG). EEG is a noninvasive electrophysiological method that records the brain's cortical electrical activity using electrodes placed over the scalp (S. P. Finnigan et al. 2004) with a high temporal resolution (Nunez and Srinivasan 2006). Among several applications, EEG is used to indicate frequency‐specific changes and to understand neurophysiological alterations in brain after ABI (Dockree and Robertson 2011; S. Finnigan and van Putten 2013). Some metrics extracted from EEG allow the identification of cognitive alterations after ABI. For instance, the power spectral analysis technique (Keser et al. 2022) in different frequency bands, such as delta (0.5–4 Hz), theta (4–8 Hz), alpha (8–12 Hz), beta (12–30 Hz), and gamma (> 30 Hz), has been used in several studies. Delta and theta waves are defined as low‐frequency activity, whereas alpha, beta, and gamma waves are mostly classified as high‐frequency activity (Brito et al. 2021).

Following a stroke, it has been shown that there is an increased activity in slow rhythms (Assenza et al. 2013) and a decrease in fast rhythms powers (Kispaeva et al. 2011; Sutcliffe et al. 2022; Yang, Shin, and Hong 2021; Zhang et al. 2023). Another study comparing stroke patients with healthy subjects revealed that alpha, theta, and delta differed significantly with lower alpha power, higher theta, and delta power for the stroke group during the resting and cognitive tasks (Hussain and Park 2021). In addition, Aminov et al. (2017) showed less theta power and more delta power during the acute phase of a stroke compared to nonstroke participants. Nevertheless, in the review of Sutcliffe et al. (2022), the authors pointed out that, although increased theta waves are frequently observed in stroke cases compared to control subjects, these findings are not systematic. After a traumatic brain injury (TBI), Franke et al. (2016) also found an increase in low‐frequency power, specifically in the right prefrontal and temporal regions. In Arciniegas' review (2011), the most consistent findings after TBI include a decrease in average alpha frequency power and a higher theta activity (Gosselin et al. 2009).

In addition to injury effect diagnosis, EEG has also been shown to be a reliable alternative method for predicting poststroke recovery (Keser et al. 2022; Vatinno et al. 2022), whether global or domain‐targeted, such as cognition. Studies have evaluated the correlations between changes in brain activity and global recovery scores such as the modified Rankin score (mRS) and the National Institutes of Health Stroke Scale (NIHSS) (Bentes et al. 2018; Rogers et al. 2020; Wu et al. 2016). In the cognitive field in particular, quantitative EEG analyses have shown that a decrease in alpha wave activity is associated with a higher risk of cognitive decline in stroke subjects both in the acute (Schleiger et al. 2014, 2017) and chronic phases (Petrovic et al. 2017; Song et al. 2015). Likewise, in TBI patients, the frontal theta band seems to be a predictive recovery marker of cognitive control (Cavanagh et al. 2020).

Several neuroimaging studies have shown that cognitive rehabilitation can lead to changes in brain activation (Galetto and Sacco 2017). According to Dockree and Robertson (2011), oscillatory activity from the EEG provides a rich source of data that offers complementary‐to‐behavioral insights into cognitive processes for a better understanding of the pathophysiological mechanisms underlying cognitive deficits.

Despite these evidence, few studies have focused on the neural modifications and neuroplastic changes induced by cognitive treatment in these populations (Galetto and Sacco 2017). Moreover, most studies using EEG aim more at diagnosis and prognosis of recovery at resting state only and focus on the acute and subacute phases post‐ABI. As a consequence, for chronic patients, there is a lack of studies investigating the mechanisms of cerebral changes (Perlstein and Larson 2011).

To rehabilitate and compensate for the deficits after ABI, cognitive intervention is offered to patients with dysexecutive syndrome (Cicerone et al. 2019 Galetto and Sacco 2017; Tomaszczyk et al. 2014). Cognitive training has been shown to be essential for shaping plasticity and enabling recovery of behavioral outcomes (Chen and D'Esposito 2010).

In the present study, a cognitive intervention, based on a combination of two approaches, has been used. First, training of EFs has been achieved using functional exercises that simulate activities of daily living on a digital support (Covirtua Cognition^1^). Along with this cognitive training, we applied high‐definition transcranial random noise electrical stimulation (HD‐tRNS) over the right dorsolateral prefrontal cortex (rDLPFC), a key region for executive functioning (Hanna‐Pladdy 2007). A 4 × 1 electrode HD‐tRNS montage has been shown to lead to higher performance improvement compared to conventional (SD) montage (i.e., two electrodes) (Chenot et al. 2022). Some studies have shown that this cortical stimulation can modulate cortical excitability (Yang, Shin, and Hong 2021), hence promoting cerebral plasticity (Elmasry, Loo, and Martin 2015; Snowball et al. 2013).

### 1.1 Research Hypothesis and Objectives

We hypothesized that the combination of cognitive training with HD‐tRNS might potentiate the effects of this rehabilitation on behavioral performance and associated changes in brain activity, or even restore activity in frequency bands that have been altered after ABI.

To evaluate the efficacy of this innovative intervention, we used a single‐case multiple study (SCED) with behavioral primary outcomes. This type of design makes it possible to evaluate the effectiveness of an intervention in a small number of patients compensated by several repeated measures.

A first part of this study focused, not presented in this article, on the analysis of behavioral data in repeated measures, assessed throughout the study by the achievement of personalized goals (GAS).

The present article focuses on secondary outcomes (EEG and TAP battery), which were only assessed twice—pre‐ and postintervention.

Our aim was therefore to explore the impact of our cognitive intervention on changes in executive functioning (TAP scores) and brain activity (EEG spectral power in the different frequency bands), at rest and while patients were performing cognitive tasks.

## 2 Methods

### 2.1 Participants

Fifteen patients were included in this study. All received clear information, and written informed consent from each patient or substitute decision maker was obtained to participate. Inclusion criteria included: (1) 20–75 year‐old, (2) sufficient understanding of the French language, (3) frontal TBI or stroke responsible for a dysexecutive syndrome as assessed by the GREFEX/GRECO battery [25], (4) affiliated to social security, and (5) signed informed consent.

Exclusion criteria were: (1) addiction (alcohol, drugs); (2) major uncorrected hearing or visual loss; (3) high blood pressure; (4) severe cardiac insufficiency; (5) uncompensated thyroid disorders; (6) major neuropsychological disorder and/or treatment targeting the central nervous system outside the treatments prescribed in the context of their pathology; (7) family or personal history of epilepsy; (8) pregnancy; (9) woman subject of childbearing without effective contraception; (10) participation in another experimental protocol involving brain stimulation within the last 4 weeks; (11) person under the protection of justice; (12) MRI contraindication; (13) refusal to be informed of a new anomaly detected during the MRI examination; and (14) other TBI or neuropsychological disorder.

### 2.2 Experimental Procedure

#### 2.2.1 Study Design

**FIGURE 1:**
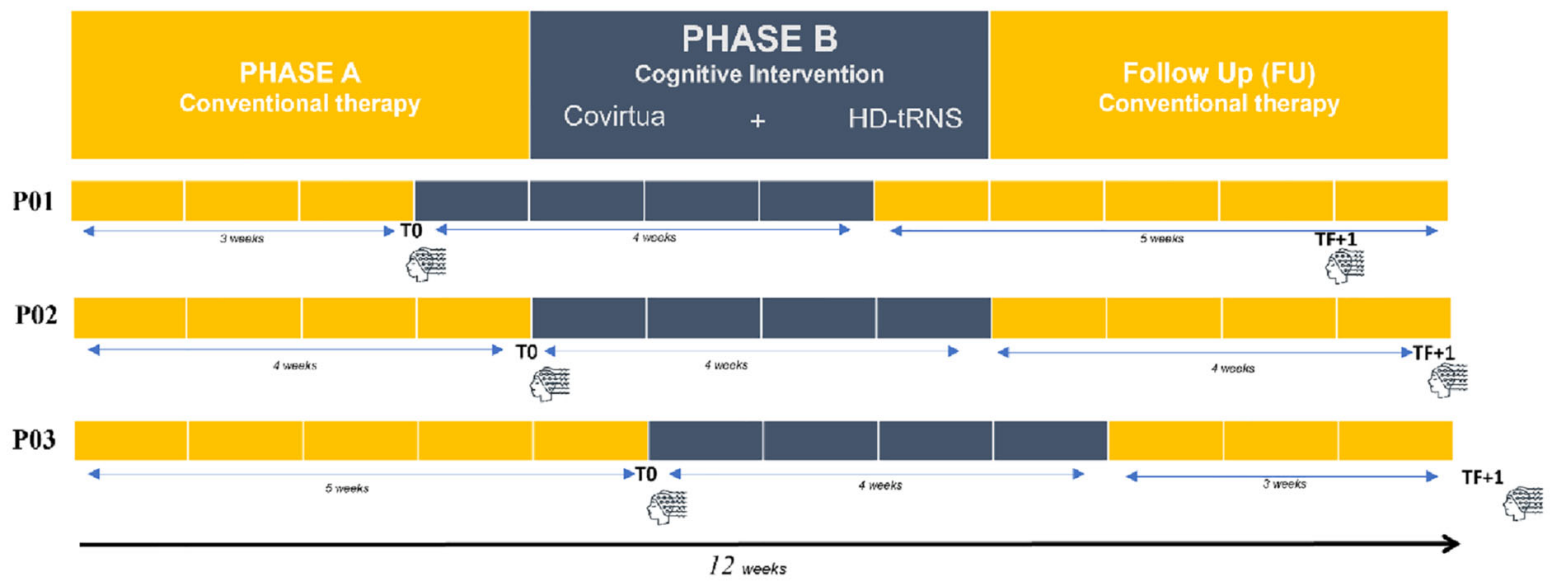
Multiple baseline design across three participants with three successive phases: Phase A: without cognitive intervention; Phase B: with an intervention combining cognitive training (Covirtua Cognition software) and transcranial random noise stimulation (tRNS), and follow‐up (FU) without cognitive intervention. The baseline duration varies for each patient between 3, 4, and 5 weeks. The intervention Phase B lasts for 4 weeks for all patients, and the total duration of the experimental procedure is 12 weeks for all participants. Each session lasts 45 min coupled with 20 min of tRNS over the right dorsolateral prefrontal cortex (DLPFC) four times a week during the first 20 min of practicing the task. Assessment visits (EEG + TAP battery) were scheduled between the Phases T0 (before the start of the intervention) and TF + 1 (1‐month follow‐up). The results of this paper only concern data acquired in T0 and TF + 1.

This study was conducted in the Department of Physical Medicine and Rehabilitation, University Hospital of Toulouse (France), over a period of 27 months. The trial was approved by the EST I ethics committee and registered at ClinicalTrials.gov (NCT04253522). A multiple‐baseline single‐case experimental design (SCED) across patients was used. An A‐B‐follow‐up (FU) paradigm with three successive phases was employed. During Phases A and FU, patients did not receive any cognitive rehabilitation. Phase B involved targeted rehabilitation, during which patients underwent four 1‐h sessions per week, for a total of 16 sessions. The term “multiple baseline” refers to varying lengths of the baseline (Phase A) across patients, meaning that the intervention was introduced sequentially and randomly across subjects, resulting in variable durations for Phases A and FU (3, 4, or 5 weeks) between patients. The duration of Phase B was consistently 4 weeks for all patients. Patients were included in groups of three and followed for 12 weeks. The study design is displayed in Figure 1.

#### 2.2.2 Treatment

##### 2.2.2.1 Cognitive Training

Covirtua software is an interactive digital medium between a therapist station (OMEN by HP) and a patient station (Microsoft Surface Pro). It proposes four close‐to‐real‐life functional activities of everyday situations in realistic environments (e.g., shopping in a supermarket) to optimize the transfer of knowledge into daily life. It also creates a “list” based on a daily‐life scenario, such as writing a shopping list to organize an event (considering the number of people and food constraints), driving in an urban environment considering road traffic, or an orientation test on a city map. These activities are offered according to patients’ abilities and adaptation (about three exercises of 10–12 min per session). The difficulty can be modulated according to the patient's performance during the exercise.

##### 2.2.2.2 Brain Stimulation (tRNS)

**FIGURE 2:**
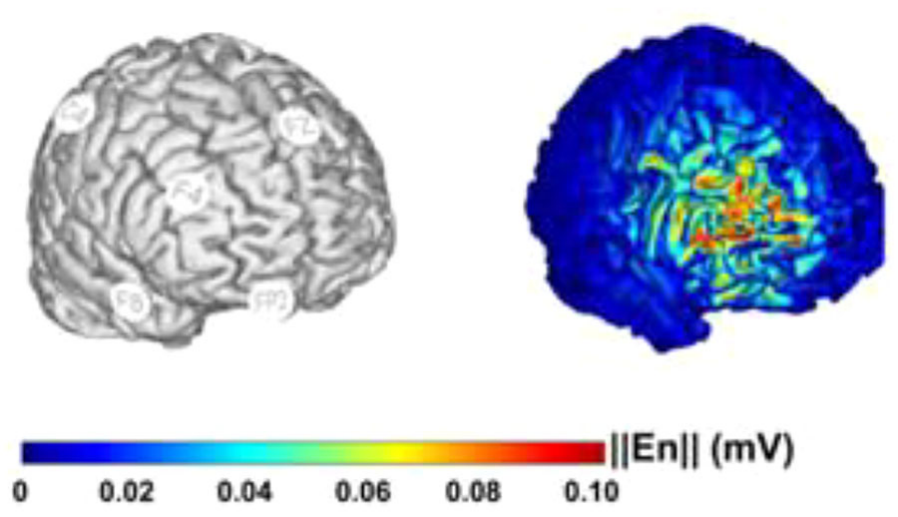
HD‐tRNS montage with theoretical influence map over the white matter of the right DLPFC. Extracted from NIC2 software v.2.0.10 (Neuroelectrics). The electrodes (NG Pistim; contact area of π cm^2^ with signal gel) were positioned on the scalp using a neoprene cap labeled with a subset of the international 10–20 EEG system. To induce a more focal stimulation, we employed a high‐definition (HD) montage that consisted of one central electrode placed over the rDLPFC at F4 as the stimulating site and four other electrodes (F8, C4, FZ, and FP2) as current return electrodes.

We opted for tRNS due to its superior efficacy compared to other stimulation methods (Fertonani, Pirulli, and Miniussi 2011). We used a HD montage that involves multiple electrodes: one central (stimulation) electrode positioned over the targeted cortical area (right DLPFC, F4) and four return electrodes (F8, C4, FZ, and FP2). It has been shown that this type of montage has the advantage of inducing a more focal stimulation compared to the conventional simple‐definition montage (i.e., two electrodes) (Villamar et al. 2013). HD‐tRNS was performed using the Neuroelectrics Starstim2 device and NIC2.0 (Neuroelectrics) software. It has been applied according to recommendations (Santarnecchi et al. 2015). While the patient performed the CoVirtua task, an oscillating current of 1 mA (−0.5 to 0.5 mA peak to peak, offset = 0) was applied at a random frequency between 100 and 500 Hz over the right dorsolateral prefrontal cortex (rDLPFC), which is directly involved in executive functions, such as processes involved in the control of complex voluntary actions (Brunyé et al. 2019) (see Figure 2).

### 2.3 Behavioral Data: Test of Attentional Performance (TAP)

Executive functions were assessed using the TAP battery (Zimmerman and Fimm 2009), which was carried out by the patients at both evaluation visits (T0 and TF + 1). Four subtests were used to assess reaction times (RTs) and correct responses (CRs) in WM, inhibition (GO), divided attention (DA), and FL. The evolution of CRs and RTs between T0 and TF + 1 was analyzed at the group level with Wilcoxon tests using RStudio (2023.09.1 + 494); effect sizes were calculated as the Z statistic (Tomczak and Tomczak, 2014) using *Wilcox_effsize* function from the *rstatix* package.

### 2.4 EEG Data

Electrophysiological measurements were also conducted at T0 and TF + 1. Evaluating these measures enabled us to determine whether the treatment effect extended beyond behavioral changes and to assess the impact of the intervention on brain activity between the beginning and the end of the intervention.

#### 2.4.1 Acquisition and Preprocessing

Electroencephalographic signals were recorded with a Biosemi Active‐two amplifier at 2048 Hz. Raw data were measured from 64 preamplified Ag‐Ag/Cl electrodes with conductive gel positioned over the head cap according to the 10–20 system.

The EEG recording was performed at rest, with closed eyes for 5 min, while the subject was seated and relaxed, and throughout the practice of the four subtests of the TAP.

Signal processing was carried out with MATLAB software (ver. R2020b), using the EEGLab toolbox (version 13.6.5b; Delorme and Makeig 2004).

EEG data were re‐referenced to the common average reference, resampled at 500 Hz, and band‐pass filtered between 0.5 and 100 Hz. An additional notch filter at 50 Hz was also applied to remove European AC line noise. Each continuous EEG was then segmented into epochs according to the condition of interest. Artifact detection and rejection were performed using independent component analyses (ICA, *Runica* with default parameters) implemented in EEGLAB. This function is a source decomposition algorithm that separates sources into independent component labels. Using the *ICLabel* function of EEGLAB, components that were classified as muscle, eye, heart, line noise, and channel noise with at least 80% confidence were subsequently rejected.

Channels with remaining aberrant waveforms were excluded and interpolated using the *EEG‐interpolate* function of EEGLAB with the “*spherical*” interpolation method (at a minimum, no electrodes were interpolated, and a maximum of three electrodes were interpolated per patient).

Finally, EEGs were not included in analyses if they were contaminated by excessive noise identified by visual inspection. All exclusions were made prior to data analysis.

#### 2.4.2 Data Analyses

EEG cleaned epochs from each experimental condition were subjected to a fast Fourier transform (FFT) to calculate power spectral values. Absolute spectral power was calculated for all electrodes, at each session and for five frequency bands: delta (0.5–4 Hz), theta (4–8 Hz), alpha (8–12 Hz), beta (12–30 Hz), and gamma (30–100 Hz). To evaluate statistical differences of spectral power bands between T0 and TF + 1, we used bootstrap statistics (Kim et al. 2018) with false discovery (FDR) correction provided by the EEGlab toolbox. FDR correction permits to maintain the Type I error at a sufficient low level (*p*‐value threshold was set to *p* < 0.05). Visualizations of these variations in each frequency band were finally done using the built‐in “plot spectra” option.

## 3 Results

### 3.1 Participants

Table 1 summarizes the demographic characteristics of each patient, including details about brain lesions and their neuropsychological profiles (e.g., Grefex score), highlighting a dysexecutive syndrome. One participant (P04) withdrew from the study due to family issues, resulting in the investigation of 14 TBI patients (7 men; 8 women; 46 y/o). Among them, five patients had experienced post‐traumatic brain injury, and 10 had poststroke conditions, with an average time since the brain lesion of 5 years.

**TABLE 1:** Participants characteristics (*n* = 14).

Participant	Age	Gender	Diagnosis time since injury (month)	Neuropsychological assessment GREFEX score_Standard deviation	
P1	56	Female	Stroke 34	Stroop (time, inter‐deno) Trail Making Test (time, B–A) Verbal fluency Categorical Letter WCST_Perseveration Number of categories Total number of errors	NF^a^ **−2.09** **−2.96** **−2.49** **−2.97** 0.30 −1.14
P2	54	Female	Stroke 33	Stroop (time, inter‐deno) Trail Making Test (time, B–A) Verbal fluency Categorical Letter WCST_Perseveration Number of categories Total number of errors	−1.5 **−4.29** **−2.85** **−2.49** **−4.79** 0.30 **−1.78**
P3	62	Female	Stroke 52	Stroop (time, inter‐deno) Trail Making Test (time, B–A) Verbal fluency Categorical Letter WCST_Perseveration Number of categories Total number of errors	0.08 0.72 **−3.03** −0.8 **−6.89** **−1.78** **−2.76**
P4	48	Male	Stroke 14	Stroop (time, inter‐deno) Trail Making Test (time, B–A) Verbal fluency Categorical Letter WCST_Perseveration Number of categories Total number of errors	0.45 −0.67 **−3.19** **−2.17** 0.66 −0.30 −0.60
P5^b^	22	Male	TBI 42	Stroop (time, inter‐deno) Trail Making Test (time, B–A) Verbal fluency Categorical Letter WCST_Perseveration Number of categories Total number of errors	0.07 0.15 **−1.73** −1.21 −0.46 0.18 0.24
P6	49	Female	Stroke 17	Stroop (time, inter‐deno) Trail Making Test (time, B–A) Verbal fluency Categorical Letter WCST_Perseveration Number of categories Total number of errors	**−2.04** 0.14 −0.78 −1.53 **−13.88** **−9.13** **−7.5**
P7	63	Male	Stroke 9	Stroop (time, inter‐deno) Trail Making Test (time, B–A) Verbal fluency Categorical Letter WCST_Perseveration Number of categories Total number of errors	NF^a^ **−2.95** **−2.92** **−3.89** **−8.41** **−2.82** **−3.33**
P8	44	Male	TBI 22	Stroop (time, inter‐deno) Trail Making Test (time, B–A) Verbal fluency Categorical Letter WCST_Perseveration Number of categories Total number of errors	**−3.66** NF^a^ −0.48 0.86 **−2.57** **−17.95** **−4.9**
P9	40	Female	Stroke 20	Stroop (time, inter‐deno) Trail Making Test (time, B–A) Verbal fluency Categorical Letter WCST_Perseveration Number of categories Total number of errors	**−7.92** **−2.73** −1.97 **−2.12** −1.4 **−2.44** **−2.33**
P10	60	Female	TBI 423	Stroop (time, inter‐deno) Trail Making Test (time, B–A) Verbal fluency Categorical Letter WCST_Perseveration Number of categories Total number of errors	0.56 0.87 0.82 −0.07 **−5.35** **−3.86** 0.66
P11^c^	20	Male	TBI 39	Stroop (time, inter‐deno) Trail Making Test (time, B–A) Verbal fluency Categorical Letter WCST_Perseveration Number of categories Total number of errors Test of Attentional Performance (TAP) (Zimmerman and Fimm 2009)^b^ Shifting: Total average time Falses Overall performance index Speed/quality ratio Inhibition: Total average time Falses Omissions Work memory: Total average time Falses Omissions Divided attention: Time omissions falses	NF^a^ NF^a^ **−1.66** NF^a^ NF^a^ NF^a^ NF^a^ 799 ms C34 12 C5^b^ −14,140 C8^b^ −8484 C16^a^ 473 ms C16^a^ 2 C34 2 C4^b^ 824 ms C38 7 C7^b^ 4 C12^b^ Auditory 718 ms C3^b^ 1 C31 Visual 761 ms C38 6 C1^b^ Total 7 C1^b^ 1 C34
P12	42	Female	Stroke 30	Stroop (time, inter‐deno) Trail Making Test (time, B–A) Verbal fluency Categorical Letter WCST_Perseveration Number of categories Total number of errors	NF^a^ **−3.97** **−3.13** **−2.86** **−4.95** **−17.9** **−8.37**
P13^b^	39	Male	Stroke 10	Stroop (time, inter‐deno) Trail Making Test (time, B–A) Verbal fluency Categorical Letter WCST_Perseveration Number of categories Total number of errors	0.42 0.5 0.01 0.71 0.67 −0.26 0.67
P14	39	Female	Stroke 21	Stroop (time, inter‐deno) Trail Making Test (time, B–A) Verbal fluency Categorical Letter WCST_Perseveration Number of categories Total number of errors	**−1.88** −1.25 −1.50 −1.19 −1.61 **−5.33** **−2.56**
P15^c^	46	Male	TBI 12	Stroop (time, inter‐deno)^c^ Trail Making Test (time, B–A) Verbal fluency Categorical Letter WCST_Perseveration Number of categories Total number of errors Test of Attentional Performance (TAP) (Zimmerman and Fimm 2009)^b^ Shifting: Total average time Falses Overall performance index Speed/quality ratio Inhibition: Total average time Falses Omissions Work memory: Total average time Falses Omissions Divided attention: Time omissions falses	NF^a^ NF^a^ NF^a^(perseveration on the same word) **−4.15** **−8.86** **−4.23** NF^a^ 564 ms C4^b^ 1 C58 0 > C14 NF^a^ Auditory 724 ms C3^b^ 1 C31 Visual 955 ms C14^b^ 0 C82 Total 1 C50 3 C12^b^

*Note*: Bold: <−1.65 SD.

^a^ NF: Not finished or not realized.

^b^ P05 and P13: Although the neuropsychological assessment scores seemed to fall within the normal range, “paper and pencil” tests may not always be sufficient to detect impaired functions. However, both patients experienced difficulties with attention and planning in their daily lives.

^c^ P11 and P15 had undergone a neuropsychological assessment using TAP's computerized test battery (Zimmerman and Fimm 2009) less than 6 months before inclusion. Therefore, the neuropsychological assessment using the Grefex battery was not repeated at the time of inclusion.

### 3.2 Behavioral Measures: TAP

**FIGURE 3:**
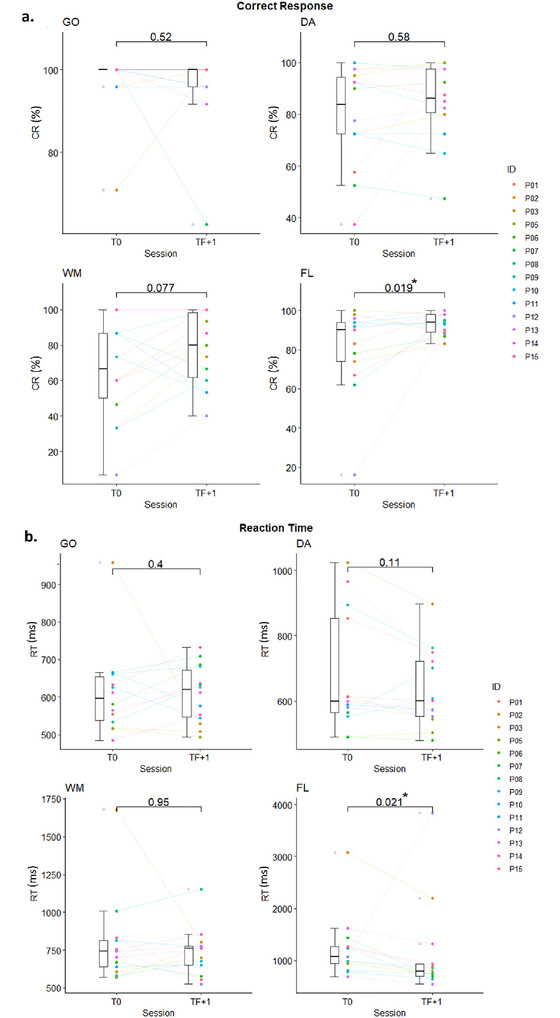
Results of the four subtests of the TAP battery in terms of CRs (a) and RTs (b) between T0 and TF + 1. *p < 0.05. The length of the box is the difference between the 75th and 25th percentiles and is called interquartile range (IQR). The upper and lower whiskers are minimum and maximum data values, excluding outliers. DA: divided attention; FL: flexibility; GO: inhibition; WM: working memory.

No significant results were observed between the two assessment times for the GO, WM, and DA subtests, either in terms of CRs or RTs. However, the results show a significant increase in CRs and a decrease in RTs for the FL subtest between T0 and TF + 1 (see Figure 3a,b). For P07, the FL test requiring sufficient bimanual motor skills to press the response buttons could not be performed due to his motor deficit. At T0, the median response time was missing for P12 WM and P07 DA due to an excessive number of false answers or omissions.

The effect size estimate of CR and RT for the FL subtest shows a moderate effect (*r* = 0.31 for CR and *r* = 0.36 for RT) between T0 and TF + 1.

### 3.3 Electrophysiological Measures: EEG Data

For the rest condition, we excluded one participant because the signal was too noisy, and four EEG datasets were excluded due to poor signal quality during cognitive tasks. Eventually, for the rest condition, 13 EEG recordings were included, nine for DA, and eight for FL, WM, and Go/conditions in the final sample.

**FIGURE 4:**
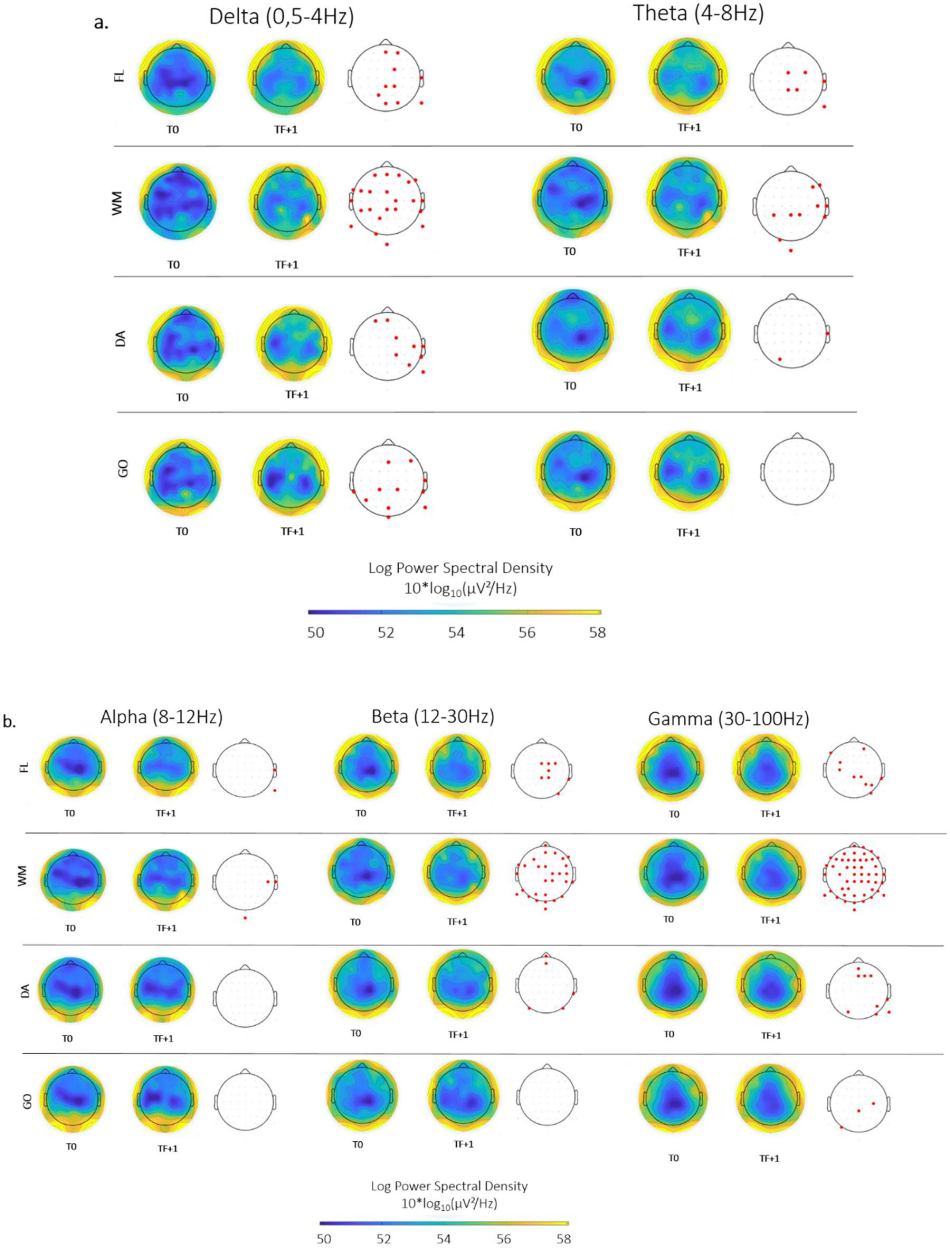
Topographical maps of the power spectrum across the participants for each frequency band of interest during the cognitive task between T0 and TF + 1. (a) Slow‐wave frequency activities. (b) Fast‐wave frequency activities. Bootstrap with FDR (*p* < 0.05). DA: divided attention; FL: flexibility; GO: inhibition; WM: working memory.

The electrodes with significantly different spectral power between T0 and TF + 1 are represented in red in Figure 4a,b.

Regarding slow‐wave activities, we observed an increase in delta wave power for all four subtests of the TAP battery. Theta waves exhibited comparable modifications except for the GO task.

Concerning fast‐wave activities, we also observed significant variations in these rhythms, with an increase in the alpha band for the FL and WM tests, an increase in the beta band for FL, WM, and AD tests, and a gamma band for all four subtests between T0 and TF + 1. For the GO, we did not observe any significant effect of the intervention in the theta, alpha, and beta frequency bands. See Table 2 for the results summary. Similarly, there were no significant variations in the EEG power spectrum for each frequency band between T0 and TF + 1 in the resting‐state condition.

**TABLE 2:** Summary of spectral power evolutions for each frequency band between T0 and TF + 1 (statistical bootstrap test with false discovery rate correction; FDR).

		Power spectrum by frequency band				
		Delta (0, 5–4 Hz)	Theta (4–8 Hz)	Alpha (8–12 Hz)	Beta (12–30 Hz)	Gamma (30–100 Hz)
Resting state		—	—	—	—	—
TEA	Flexibility	↗	↗	↗	↗	↗
	Working memory	↗	↗	↗	↗	↗
	Divided attention	↗	↗	—	↗	↗
	GoNoGo	↗	—	—	—	↗

The arrows indicate a significant increase in spectral power (*p* < 0.05) on at least one electrode.

Overall, after the cognitive intervention (T0 vs. TF + 1), analyses of EEG data show an increase in slow (delta and theta) and fast (alpha, beta, and gamma) waves solely during the practice of the cognitive task (TAP).

## 4 Discussion

### 4.1 Main Results

ABI leads to changes in cerebral activity and deficits in executive functioning. To compensate for these deficits, patients can benefit from a cognitive intervention. The purpose of the present study was to assess the impact of a cognitive intervention on pre‐ and postcognitive task performance and related cerebral activity. We hypothesized that cognitive intervention would induce changes in brain activity and improve executive functioning.

Regarding EFs, the results over the TAP battery demonstrate that cognitive training, combined with tRNS over the right DLPFC, mainly induced significant improvements in mental FL.

From an electrophysiological point of view, except for the alpha band while practicing the DA task and the theta, alpha, and beta bands during the GO task, our results relate that the cognitive intervention had an impact on all frequency bands during cognitive task performance.

After ABI, an increase in slow‐wave activities and a decrease in fast‐wave activities have been observed (Sutcliffe et al. 2022). Following an intervention, one would expect a restoration of these intrinsic parameters, that is, a decrease in slow‐wave and an increase in fast‐wave powers.

In the present study, however, although we did observe an increase in fast‐wave activities (alpha, beta, and gamma), we also found an a priori counterintuitive increase in slow‐wave activity (delta and theta) after the intervention.

It has been demonstrated that theta oscillations are observed during various cognitive tasks, with the anterior cingulate cortex as a key generator, an integral component of networks—including the rDLPFC—supporting cognitive processes (Mitchell et al. 2008; Cavanagh et al. 2014). The postintervention increase in theta and delta wave powers could thus be interpreted as a reinforcement of the compensatory mechanisms that took place after the lesion (Liu Sinan et al. 2021), hence facilitating task performance. Moreover, these oscillations are considered to orchestrate executive functioning in the prefrontal cortex (Cavanagh et al. 2020) and may lead to an increase in fast‐wave activity, which could have also been achieved thanks to cross‐frequency coupling mechanisms (Canolty et al. 2006).

For the fast‐wave–related results, we can assume that the intervention induced restoration mechanisms in the alpha, beta, and gamma frequency bands. However, it should be noted that these brain changes are accompanied by an improvement in mental FL performance only. No significant results were observed between each assessment time for the GO, WM, and DA subtests, either in terms of percentage of CR or RT. As a consequence, these neurophysiological modifications do not appear to be sufficient to improve performance in these functions. In addition, for the GO test, a ceiling effect has been observed, which may have limited the detection of potential improvements in performance if any.

Finally, we did not observe any significant difference in spectral power at rest whatever the frequency band, which is an argument in favor of the specificity of our intervention during cognitive tasks.

Overall, after a brain injury, patients may require more resources to perform a cognitive task, necessitating an increase in the investigated brain wave activity. Cognitive intervention could lead to a readjustment of the mechanisms involved in the recovery process that is observable as a global spectral power increase.

### 4.2 Limits

The major limitation of our study is due to the assessment of EEG and TAP performance in a group‐level pre–postintervention design in a small sample size. In SCED, however, the sample size is tailored to conduct robust statistical analyses of the repeated measure; the secondary criteria however are not robust enough for the small sample size. This limitation restricts the possibility of in‐depth statistical analyses, as we lack sufficient data to establish reliable and robust correlations between behavioral performances and observed changes in spectral power within frequency bands. In addition, it is difficult to assert with certainty that the observed effects are due to the cognitive intervention. Finally, despite a specific effect for the FL task and not for the others or the rest condition, it is important to stress that the absence of a control group does not allow us to exclude the effect of time on performance improvement and brain changes.

### 4.3 Perspectives and Clinical Application

In view of these limitations, the first step would be to validate these EEG biomarkers as reliable enough to predict effective rehabilitation outcomes with a larger cohort of patients. In parallel, it would be relevant to use these biomarkers as repeated measures in a SCED.

Indeed, SCEDs are suitable to evaluate the effect of a cognitive intervention. The most common way to assess it is to use behavioral tasks, however, which may be sensitive to certain biases (test–retest effect or influence of external factors) and mask or, on the contrary, amplify the real effectiveness of the intervention.

Furthermore, in the context of cognitive training strategy, behavioral effects may not be detectable until sometime after the intervention ends. Therefore, it would be interesting to dispose of brain markers that (1) can predict cognitive recovery (Tahmi et al. 2022), (2) can detect functional changes earlier, and (3) are less sensitive to the issue of motor output, often associated with cognitive impairments in this population.

Moreover, significant EEG results without behavioral improvement could also help in refining the selection of behavioral tests by directing toward tests that are more sensitive to changes while being less sensitive to ceiling or floor effects, or toward tests that assess executive functions in a less isolated manner (i.e., more ecological).

We are convinced of the value of SCED and its “person‐centered” approach in such a heterogeneous population with diverse symptoms and varying recovery trajectories. The choice of electrophysiological measurements as repeated measures would also make it possible to explore the cerebral reorganization that could be specific to each patient (Green 2003). Combining behavioral and electrophysiological assessments seems to be a promising approach to enhance the quality of studies aiming at assessing the effectiveness of cognitive intervention, thereby improving the reliability of the results. This preliminary study paves the way for the use of new methods to measure the effects and mechanisms of interventions, enabling the development of personal interventions (Chen and D'Esposito 2010), increasing the effectiveness of existing therapies, and providing insights into the underlying neuroplastic process (Keser et al. 2022).

Further research in this field is crucial for (1) gaining a better understanding of the characteristics of the neural activity induced by cognitive intervention, (2) establishing correlations between electrophysiological results and behavioral data, and (3) deepening the value and potential application of EEG measurements as reliable biomarkers to assess the effectiveness of cognitive intervention, in association with clinical behavioral assessments.

## 5 Conclusion

Our study investigated the impact of a cognitive intervention on EEG changes and executive function impairments in patients with dysexecutive syndrome following an ABI in the chronic phase.

Our findings suggest that our cognitive intervention could have led to an improvement in mental FL associated with an increase in brain activity across both slow and fast cortical frequency bands activity during a cognitive task. EEG proves to be a sensitive tool for monitoring brain activity. We suggest that it could be used as a repeated measure in SCED, complementing behavioral assessments, to evaluate the effectiveness of a cognitive intervention.

Further research in electrophysiology, specifically examining changes in brain activity associated with cognitive interventions, will contribute to advancing intervention strategies and optimizing the care management of patients with brain injuries.

## Author Contributions

**C. Lebely**: Writing–original draft, conceptualization, investigation, methodology, visualization, writing–review and editing, software, project administration. **E. Lepron**: Conceptualization, investigation, methodology, visualization, software, writing–review and editing. **I. Bigarre**: Writing–review and editing, validation. **C. Hamery**: Methodology, software, data curation. **X. De Boissezon**: Funding acquisition, writing–review and editing, writing–original draft, supervision, data curation. **S. Scannella**: Funding acquisition, writing–review and editing, writing–original draft, supervision, software, methodology.

## Conflicts of Interest

The authors declare no conflicts of interest.

### Peer Review

The peer review history for this article is available at https://publons.com/publon/10.1002/brb3.70148.

## Data Availability

The data that support the findings of this study are available from the corresponding author upon reasonable request.

## Endnotes

1
https://www.covirtua.com

## References

[brb370148-bib-0001] Aminov, A. , J. M. Rogers , S. J. Johnstone , S. Middleton , and P. H. Wilson . 2017. “Acute Single Channel EEG Predictors of Cognitive Function After Stroke.” PLoS ONE 12, no. 10: e0185841. 10.1371/journal.pone.0185841. 28968458 PMC5624638

[brb370148-bib-0002] Arciniegas, D. B. 2011. “Clinical Electrophysiologic Assessments and Mild Traumatic Brain Injury: State‐of‐the‐Science and Implications for Clinical Practice.” International Journal of Psychophysiology 82, no. 1: 41–52. 10.1016/j.ijpsycho.2011.03.004. 21419178

[brb370148-bib-0003] Assenza, G. , F. Zappasodi , P. Pasqualetti , F. Vernieri , and F. Tecchio . 2013. “A Contralesional EEG Power Increase Mediated by Interhemispheric Disconnection Provides Negative Prognosis in Acute Stroke.” Restorative Neurology and Neuroscience 31, no. 2: 177–188. 10.3233/RNN-120244. 23254689

[brb370148-bib-0004] Bentes, C. , A. R. Peralta , P. Viana , et al. 2018. “Quantitative EEG and Functional Outcome Following Acute Ischemic Stroke.” Clinical Neurophysiology 129, no. 8: 1680–1687. 10.1016/j.clinph.2018.05.021. 29935475

[brb370148-bib-0005] Brito, R. , A. Baltar , M. Berenguer‐Rocha , et al. 2021. “Intrahemispheric EEG: A New Perspective for Quantitative EEG Assessment in Poststroke Individuals.” Neural Plasticity 2021: 1–8. 10.1155/2021/5664647. PMC8481048 34603441

[brb370148-bib-0006] Brunyé, T. T. , E. K. Hussey , E. B. Fontes , and N. Ward . 2019. “Modulating Applied Task Performance via Transcranial Electrical Stimulation.” Frontiers in Human Neuroscience 13: 140. 10.3389/fnhum.2019.00140. 31114491 PMC6503100

[brb370148-bib-0052] Canolty, R. T. , E. Edwards , S. S. Dalal , et al. 2006. “High Gamma Power Is Phase‐locked to Theta Oscillations in human Neocortex.” Science (New York, N.Y.) 313, no. 5793: 1626–1628. 10.1126/science.1128115. 16973878 PMC2628289

[brb370148-bib-0050] Cavanagh, J. F. , and M. J. Frank . 2014. “Frontal Theta as a Mechanism for Cognitive Control.” Trends in Cognitive Sciences 18, no. 8: 414–421. 10.1016/j.tics.2014.04.012. 24835663 PMC4112145

[brb370148-bib-0007] Cavanagh, J. F. , R. E. Rieger , J. K. Wilson , et al. 2020. “Joint Analysis of Frontal Theta Synchrony and White Matter Following Mild Traumatic Brain Injury.” Brain Imaging and Behavior 14, no. 6: 2210–2223. 10.1007/s11682-019-00171-y. 31368085 PMC6992511

[brb370148-bib-0008] Chen, A. J.‐W. , and M. D'Esposito . 2010. “Traumatic Brain Injury: From Bench to Bedside to Society.” Neuron 66, no. 1: 11–14. 10.1016/j.neuron.2010.04.004. 20399725

[brb370148-bib-0048] Chenot, Q. , C. Hamery , E. Lepron , et al. 2022. “Performance After Training in a Complex Cognitive Task Is Enhanced by High‐definition Transcranial Random Noise Stimulation.” Scientific Reports 12, no. 1: 4618. 10.1038/s41598-022-08545-x. 35301388 PMC8931133

[brb370148-bib-0046] Cicerone, K. D. , Y. Goldin , K. Ganci , et al. 2019. “Evidence‐Based Cognitive Rehabilitation: Systematic Review of the Literature from 2009 through 2014.” Archives of Physical Medicine and Rehabilitation 100, no. 8: 1515–1533. 10.1016/j.apmr.2019.02.011. 30926291

[brb370148-bib-0009] Clark, L. , and F. Manes . 2004. “Social and Emotional Decision‐Making Following Frontal Lobe Injury.” Neurocase 10, no. 5: 398–403. 10.1080/13554790490882799. 15788279

[brb370148-bib-0049] Delorme, A. , and S. Makeig . 2004. “EEGLAB: an Open Source Toolbox for Analysis of Single‐trial EEG Dynamics Including Independent Component Analysis.” Journal of Neuroscience Methods 134, no. 1: 9–21. 10.1016/j.jneumeth.2003.10.009. 15102499

[brb370148-bib-0010] Dockree, P. M. , and I. H. Robertson . 2011. “Electrophysiological Markers of Cognitive Deficits in Traumatic Brain Injury: A Review.” International Journal of Psychophysiology 82, no. 1: 53–60. 10.1016/j.ijpsycho.2011.01.004. 21238506

[brb370148-bib-0011] Elmasry, J. , C. Loo , and D. Martin . 2015. “A Systematic Review of Transcranial Electrical Stimulation Combined With Cognitive Training.” Restorative Neurology and Neuroscience 33, no. 3: 263–278. 10.3233/RNN-140473. 25624425

[brb370148-bib-0012] Fertonani, A. , C. Pirulli , and C. Miniussi . 2011. “Random Noise Stimulation Improves Neuroplasticity in Perceptual Learning.” Journal of Neuroscience 31, no. 43: 15416–15423. 10.1523/JNEUROSCI.2002-11.2011. 22031888 PMC6703532

[brb370148-bib-0013] Finnigan, S. , and M. van Putten . 2013. “EEG in Ischaemic Stroke: Quantitative EEG Can Uniquely Inform (Sub‐)Acute Prognoses and Clinical Management.” Clinical Neurophysiology 124, no. 1: 10–19. 10.1016/j.clinph.2012.07.003. 22858178

[brb370148-bib-0014] Finnigan, S. P. , S. E. Rose , M. Walsh , et al. 2004. “Correlation of Quantitative EEG in Acute Ischemic Stroke With 30‐Day NIHSS Score: Comparison With Diffusion and Perfusion MRI.” Stroke 35, no. 4: 899–903. 10.1161/01.STR.0000122622.73916.d2. 15001786

[brb370148-bib-0015] Franke, L. M. , W. C. Walker , K. W. Hoke , and J. R. Wares . 2016. “Distinction in EEG Slow Oscillations Between Chronic Mild Traumatic Brain Injury and PTSD.” International Journal of Psychophysiology 106: 21–29. 10.1016/j.ijpsycho.2016.05.010. 27238074

[brb370148-bib-0016] Galetto, V. , and K. Sacco . 2017. “Neuroplastic Changes Induced by Cognitive Rehabilitation in Traumatic Brain Injury: A Review.” Neurorehabilitation and Neural Repair 31, no. 9: 800–813. 10.1177/1545968317723748. 28786307

[brb370148-bib-0017] Godefroy, O. , P. Azouvi , P. Robert , M. Roussel , D. LeGall , and T. Meulemans . 2010. “Dysexecutive Syndrome: Diagnostic Criteria and Validation Study.” Annals of Neurology 68, no. 6: 855–864. 10.1002/ana.22117. 21194155

[brb370148-bib-0018] Gosselin, N. , M. Lassonde , D. Petit , et al. 2009. “Sleep Following Sport‐Related Concussions.” Sleep Medicine 10, no. 1: 35–46. 10.1016/j.sleep.2007.11.023. 18226956

[brb370148-bib-0019] Green, J. B. 2003. “Brain Reorganization After Stroke.” Topics in Stroke Rehabilitation 10, no. 3: 1–20. 10.1310/H65X-23HW-QL1G-KTNQ. 14681816

[brb370148-bib-0020] Hanna‐Pladdy, B. 2007. “Dysexecutive Syndromes in Neurologic Disease.” Journal of Neurologic Physical Therapy 31, no. 3: 119–127. 10.1097/NPT.0b013e31814a63c2. 18025957

[brb370148-bib-0021] Hussain, I. , and S.‐J. Park . 2021. “Quantitative Evaluation of Task‐Induced Neurological Outcome After Stroke.” Brain Sciences 11, no. 7: 900. 10.3390/brainsci11070900. 34356134 PMC8307254

[brb370148-bib-0022] Kennedy, M. R. T. , C. Coelho , L. Turkstra , et al. 2008. “Intervention for Executive Functions After Traumatic Brain Injury: A Systematic Review, Meta‐Analysis and Clinical Recommendations.” Neuropsychological Rehabilitation 18, no. 3: 257–299. 10.1080/09602010701748644. 18569745

[brb370148-bib-0023] Keser, Z. , S. C. Buchl , N. A. Seven , et al. 2022. “Electroencephalogram (EEG) With or Without Transcranial Magnetic Stimulation (TMS) as Biomarkers for Post‐Stroke Recovery: A Narrative Review.” Frontiers in Neurology 13: 827866. 10.3389/fneur.2022.827866. 35273559 PMC8902309

[brb370148-bib-0024] Kim, S.‐E. , D. Ba , and E. N. Brown . 2018. “A Multitaper Frequency‐Domain Bootstrap Method.” IEEE Signal Processing Letters 25, no. 12: 1805–1809. 10.1109/lsp.2018.2876606. 32002009 PMC6990459

[brb370148-bib-0025] Kispaeva, T. T. , I. V. Kichuk , I. M. Shetova , et al. 2011. “Clinical‐Electrophysiological Characteristics of the Cognitive Sphere in Patients in the Acute Period of the First Cerebral Ischemic Stroke.” Zhurnal Nevrologii I Psikhiatrii Imeni S.S. Korsakova 111, no. 2 pt. 8: 25–30. 22224241

[brb370148-bib-0051] Liu, S. , C. Shi , X. Ma , B. Zhao , X. Chen , and L. Tao . 2021. “Cognitive Deficits and Rehabilitation Mechanisms in Mild Traumatic Brain Injury Patients Revealed by EEG Connectivity Markers.” Clinical Neurophysiology: Official Journal of the International Federation of Clinical Neurophysiology 132, no. 2: 554–567. 10.1016/j.clinph.2020.11.034. 33453686

[brb370148-bib-0026] Mitchell, D. J. , N. McNaughton , D. Flanagan , and I. J. Kirk . 2008. “Frontal‐Midline Theta From the Perspective of Hippocampal “Theta”.” Progress in Neurobiology 86, no. 3: 156–185. 10.1016/j.pneurobio.2008.09.005. 18824212

[brb370148-bib-0027] Nunez, P. L. , and R. Srinivasan . 2006. Electric Fields of the Brain: The Neurophysics of EEG. New York: Oxford University Press.

[brb370148-bib-0028] Perlstein, W. M. , and M. J. Larson . 2011. “Psychophysiology and Brain Imaging of Cognition and Affect Following Traumatic Brain Injury: An Overview of the Special Issue.” International Journal of Psychophysiology 82, no. 1: 1–3. 10.1016/j.ijpsycho.2011.07.013. 21820016

[brb370148-bib-0029] Petrovic, J. , V. Milosevic , M. Zivkovic , et al. 2017. “Slower EEG Alpha Generation, Synchronization and “Flow”—Possible Biomarkers of Cognitive Impairment and Neuropathology of Minor Stroke.” PeerJ 5: e3839. 10.7717/peerj.3839. 28970969 PMC5623310

[brb370148-bib-0030] Rabinowitz, A. R. , and H. S. Levin . 2014. “Cognitive Sequelae of Traumatic Brain Injury.” Psychiatric Clinics of North America 37, no. 1: 1–11. 10.1016/j.psc.2013.11.004. 24529420 PMC3927143

[brb370148-bib-0031] Rogers, J. , S. Middleton , P. H. Wilson , and S. J. Johnstone . 2020. “Predicting Functional Outcomes After Stroke: An Observational Study of Acute Single‐Channel EEG.” Topics in Stroke Rehabilitation 27, no. 3: 161–172. 10.1080/10749357.2019.1673576. 31707947

[brb370148-bib-0032] Santarnecchi, E. , A.‐K. Brem , E. Levenbaum , T. Thompson , R. C. Kadosh , and A. Pascual‐Leone . 2015. “Enhancing Cognition Using Transcranial Electrical Stimulation.” Current Opinion in Behavioral Sciences 4: 171–178. 10.1016/j.cobeha.2015.06.003.

[brb370148-bib-0033] Schleiger, E. , N. Sheikh , T. Rowland , A. Wong , S. Read , and S. Finnigan . 2014. “Frontal EEG Delta/Alpha Ratio and Screening for Post‐Stroke Cognitive Deficits: The Power of Four Electrodes.” International Journal of Psychophysiology 94, no. 1: 19–24. 10.1016/j.ijpsycho.2014.06.012. 24971913

[brb370148-bib-0034] Schleiger, E. , A. Wong , S. Read , T. Rowland , and S. Finnigan . 2017. “Poststroke QEEG Informs Early Prognostication of Cognitive Impairment.” Psychophysiology 54, no. 2: 301–309. 10.1111/psyp.12785. 28118690

[brb370148-bib-0035] Snowball, A. , I. Tachtsidis , T. Popescu , et al. 2013. “Long‐Term Enhancement of Brain Function and Cognition Using Cognitive Training and Brain Stimulation.” Current Biology 23, no. 11: 987–992. 10.1016/j.cub.2013.04.045. 23684971 PMC3675670

[brb370148-bib-0036] Song, J. , C. Davey , C. Poulsen , et al. 2015. “EEG Source Localization: Sensor Density and Head Surface Coverage.” Journal of Neuroscience Methods 256: 9–21. 10.1016/j.jneumeth.2015.08.015. 26300183

[brb370148-bib-0037] Sutcliffe, L. , H. Lumley , L. Shaw , R. Francis , and C. I. Price . 2022. “Surface Electroencephalography (EEG) During the Acute Phase of Stroke to Assist With Diagnosis and Prediction of Prognosis: A Scoping Review.” BMC Emergency Medicine 22, no. 1: 29. 10.1186/s12873-022-00585-w. 35227206 PMC8883639

[brb370148-bib-0038] Tahmi, M. , V. A. Kane , M. A. Pavol , and I. A. Naqvi . 2022. “Neuroimaging Biomarkers of Cognitive Recovery After Ischemic Stroke.” Frontiers in Neurology 13: 923942. 10.3389/fneur.2022.923942. 36588894 PMC9796574

[brb370148-bib-0047] Tomaszczyk, J. C. , N. L. Green , D. Frasca , et al. 2014. “Negative Neuroplasticity in Chronic Traumatic Brain Injury and Implications for Neurorehabilitation.” Neuropsychology Review 24, no. 4: 409–427. 10.1007/s11065-014-9273-6. 25421811 PMC4250564

[brb370148-bib-0039] van der Flier, W. M. , I. Skoog , J. A. Schneider , et al. 2018. “Vascular Cognitive Impairment.” Nature Reviews. Disease Primers 4: 18003. 10.1038/nrdp.2018.3. 29446769

[brb370148-bib-0040] Vatinno, A. A. , A. Simpson , V. Ramakrishnan , H. S. Bonilha , L. Bonilha , and N. J. Seo . 2022. “The Prognostic Utility of EEG in Stroke Recovery: A Systematic Review and Meta‐Analysis.” Neurorehabilitation and Neural Repair 36, no. 4–5: 255–268. 10.1177/15459683221078294. 35311412 PMC9007868

[brb370148-bib-0041] Villamar, M. F. , M. S. Volz , M. Bikson , A. Datta , A. F. Dasilva , and F. Fregni . 2013. “Technique and Considerations in the Use of 4 × 1 Ring High‐Definition Transcranial Direct Current Stimulation (HD‐tDCS).” Journal of Visualized Experiments 77: e50309. 10.3791/50309. PMC3735368 23893039

[brb370148-bib-0042] Wu, J. , R. Srinivasan , E. Burke Quinlan , A. Solodkin , S. L. Small , and S. C. Cramer . 2016. “Utility of EEG Measures of Brain Function in Patients With Acute Stroke.” Journal of Neurophysiology 115, no. 5: 2399–2405. 10.1152/jn.00978.2015. 26936984 PMC4922461

[brb370148-bib-0043] Yang, D. , Y.‐I. Shin , and K.‐S. Hong . 2021. “Systemic Review on Transcranial Electrical Stimulation Parameters and EEG/fNIRS Features for Brain Diseases.” Frontiers in Neuroscience 15: 629323. 10.3389/fnins.2021.629323. 33841079 PMC8032955

[brb370148-bib-0044] Zhang, J. J. , D. I. Sánchez Vidaña , J. N.‐M. Chan , et al. 2023. “Biomarkers for Prognostic Functional Recovery Poststroke: A Narrative Review.” Frontiers in Cell and Developmental Biology 10: 1062807. 10.3389/fcell.2022.1062807. 36699006 PMC9868572

[brb370148-bib-0045] Zimmerman, P. , and B. Fimm . 2009. “Test d'évaluation de l'attention. Version 2.1.” https://www.psytest.net/fr/tests/tap‐tests‐d‐evaluation‐de‐l‐attention/configuration‐requise.

